# 

**Published:** 1840-04-01

**Authors:** 


### BIBLIOGRAPHICAL RECORD.

MR. VAN VOORST'S PUBLICATIONS.

1. We omitted to notice in the Bibliographical Review, the publications of this enterprising bookseller. There lie before us Parts XV. XVI. & XVII. of the History of British Birds, by WILLIAM YARRELL—and Parts VIII. and IX. of a General Outline of the Animal Kingdom, by THOMAS RYMER JONES. These parts sustain the character of their predecessors, and give additional assurance to those who have purchased the earlier numbers. We cordially recommend them,

2. Observations on the Disorders of the General Health of Females, called CHLOROSIS; shewing the true Cause to be entirely independent of Peculiarities of Sex. By SAMUEL FOX, Surgeon. Octavo, pp. 132. Highley, Dec. 1839.

3. The Physiognomy of Mental Diseases. By Sir ALEX. MORISON, M.D. No. 17. Imbecility. Price 3s. 6d. Jan. 1840.

4. Observations on Disease. By CHAS. LENDRICK, M. D. Octavo, pp. 23. Dublin, 1839.

5. Medical Portrait Gallery. No. 23. Price 3s. By Mr. PETTIGREW. Memoir and Portrait of Galen and of G. J. Guthrie, Esq.

6. The Cyclopædia of Practical Surgery. Edited by W. B. COSTELLO, M.D. &c. &c. Illustrated with Wood-cuts. Part IV. Jan. 1840. Price 5s. Sherwood and Co. 1840.

7. *Phrenology.* Correspondence between the Rev. Sir HENRY THOMPSON, Bart. and W. C. ENGLEDICE, M.D. Letter 1, price One Shilling. Letter 2, price One Penny. Letter 3, price Threepence. Portsmouth, 1839.

8. The intimate Structure of the Secretory Glands. By J. MULLER, M.D. Translated by S. SOLLY, F.R.S. With 4 Plates. Butler, London, 1840.

9. Annals of the London Homœopathic Dispensary, No. 31, Ely Place, Holborn. Physician, Dr. Curie. Jan. 1840.

10. A History of British Birds. By W. YARRELL, F.L.S. &c. Part XVI. Price 2s. 6d. Van Voorst, Paternoster Row. Jan. 1840.

11. The Eye. By WILLIAM JEAFFERSON, Esq. late Superintendant Surgeon of the Bombay Eye Infirmary, in the Hon. East India Company's Service. Illustrated by Official Reports of 53,359 Ophthalmic Cases, treated by him in that Institution. Renshaw, Strand. 1840.

12. Observations on the Climate of New Zealand; principally with reference to its Sanative Character. By WILLIAM SWAINSON, Esq. Octavo, pp. 80. Smith and Elder, 1840.

13. The Principles of Botany, structural, functional, and systematic, condensed and immediately adapted to the Use of Medical Students. By W. HUGHES WILLSHIRE, M.D. Lecturer on Botany at the Charing Cross Hospital. Octavo, pp. 230. Highley, London. 1840.

☞ *This class-book is very well adapted to the student in medicine.*

14. Animal Mechanism and Physiology. By JOHN H. GRISCOM, M.D. (One of the volumes of Harper's Family Library, No. 85.) New York, 1839.

☞ *This Treatise is exceedingly well calculated to render the interesting subject of animal mechanism familiar to the general reader.*

15. Twenty-fifth and Twenty-sixth Annual Report of the Directors of the Glasgow Royal Asylum for Lunatics. Jan. 1839. and Jan. 1840.

16. Elements of the Practice of Medicine. By CHARLES LENDRICK, M.D. Queen's Professor of the Practice of Medicine, &c. Part I. Dublin, 1840. Price 3s.

17. The Physiognomy of Mental Diseases. By Sir ALEX. MORISON, M.D. No. 18. Imbecility. Price 3s. 6d.

☞ *This Fasciculus contains six portraits.*

18. The British Temperance Advocate and Journal. Published under the Authority of the British Association for the Promotion of Temperance. Vol. II. No. 2. Feb. 15, 1840. Price 1½d. Douglas.

☞ *We wish this meritorious publication and attempt every success.*

---

19. A Manual of the Bowels, and the Treatment of their principal Disorders from Infancy to Old Age. By JAMES BLACK, M.D. Small octavo, pp. 240. Longman and Co. 1840.

---

20. The Naturalist's Library. Conducted by Sir WILLIAM JARDINE, Bart. Entomology. Bees. Edinb. and London. 1840.

---

21. A Treatise on the Ear; including its Anatomy, Physiology, and Pathology. For which the Author obtained a Gold Medal in the University of Edinburgh. By Jos. WILLIAMS, M.D. Member of the Royal College of Surgeons. Octavo, pp. 244. With Plates. Churchill, London, 1840.

---

22. A Compendium of Materia Medica and Pharmacy, adapted to the London Pharmacopœia; embodying all the New French, American, and Indian Medicines. And also comprising a Summary of Practical Toxicology. By HUNTER LANE, M.D. &c. &c. Duodecimo, pp. 308. Churchill. Feb. 1840.

☞ *A very meritorious work.*

---

23. Aphorisms of the Treatment and Management of the Insane; with Considerations on Public and Private Lunatic Asylums, pointing out the Errors in their present System. By J. G. MILLINGEN, M.D. Surgeon to the Forces, late Medical Superintendent of the County of Middlesex Lunatic Asylum at Hanwell, &c. Duodecimo, pp. 202. Churchill, London. Feb. 1840.

---

24. Vues Generales sur l'Etude Scientifique et Pratique des Deformités du Systeme Osseux, &c. Par le Docteur JULES GUERIN, Directeur de l'Institut Orthopedique, &c. Paris, 1840.

---

25. Memoire sur l'Extension Segmoide et la Flexion, &c. Par M. GUERIN. 1840.

26. Memoire sur les Caracteres Generaux du Rachelisme, &c. Par le Docteur GUERIN. Paris, 1840.

---

27. Memoires sur les Varietés Anatomiques du Pied-Bot Congenital, &c. Par le Docteur GUERIN. Paris, 1840.

---

28. Memoire sur l'Etiologie Generale des Pieds-Bots Congenitaux, &c. Par le Docteur GUERIN. Paris, 1840.

---

29. Memoire sur les Deviations Simulées de la Colonne Vertebrale, &c. Par M. GUERIN. Paris, 1840.

---

30. Memoire sur Nouvelle Methode de Traitement des Torticolis Ancien. Par M. GUERIN. Paris, 1840.

---

31. Pathological Observations on the Diseases of the Uterus. By ROBERT LEE, M.D. F.R.S. The coloured Illustrations from original Drawings by Mr. Perry. Part I. Price 14s. Churchill. 1840. Four coloured Plates.

---

32. On the Influence of Artificial Light in causing impaired Vision; and on some Methods of preventing or lessening its injurious Effects on the Eye. By JAMES HUNTER, M.D. Surgeon to the Eye Dispensary of Edinburgh. Laing and Forbes, Ed. Octavo, pp. 96. With plates. Price 3s.

---

33. Rudiments of Animal Physiology. For use in Schools, and for private Instruction. By Dr. G. HAMILTON, of Falkirk. (Chambers' Educational Course.) Octavo, pp. 104. W. & R. Chambers, Ed. 1840.

☞ *This little volume is extremely well calculated to convey sufficient information to the general reader on the various points of structure and function in the animal economy of man in particular.*

---

34. Transactions of the Medical and Physical Society of Bombay. Vol. II. 1840.

---

35. Practical Observations on Distortions of the Spine, Chest, and Limbs; together with remarks on paralytic and other diseases connected with impaired or defective motion. By WILLIAM TILLEARD WARD, F.L.S. 8vo. pp. 202. Renshaw, Strand, 1840.

